# Acupoint Catgut Embedding Improves the Lipopolysaccharide-Induced Acute Respiratory Distress Syndrome in Rats

**DOI:** 10.1155/2020/2394734

**Published:** 2020-05-02

**Authors:** Dan Li, Tian Sun, Laiting Chi, Dengming Zhao, Wenzhi Li

**Affiliations:** ^1^Department of Anesthesiology, Second Affiliated Hospital of Harbin Medical University, Harbin 150086, China; ^2^Heilongjiang Province Key Lab of Research on Anesthesiology and Critical Care Medicine, Harbin 150086, China; ^3^Qiqihar Hospital of Traditional Chinese Medicine, Qiqihar 161000, China

## Abstract

**Background:**

This study investigated the potential therapeutic effects of acupoint catgut embedding (ACE) at ST36 and BL13 on lipopolysaccharide- (LPS-) induced acute respiratory distress syndrome (ARDS) in rats.

**Materials and Methods:**

Male Sprague-Dawley rats were randomized into the normal saline (NS group with a sham procedure), lipopolysaccharide (LPS group with a sham procedure), and LPS plus ACE (LPS+ACE with ACE at bilateral BL13 and ST36 acupoints one day before LPS injection) groups. After intratracheal instillation of normal saline or LPS (0.5 mg/kg), all rats were subjected to mechanical ventilation for 4 h. Their blood gas was analyzed before and after lung injury, and their lung pressure-volumes were measured longitudinally. The levels of TNF-*α*, IL-6, IL-10, and phosphatidylcholine (PC) and total proteins (TP) in bronchial alveolar lavage fluid (BALF) were assessed. Their wet to dry lung weight ratios, histology, myeloperoxidase (MPO), superoxide dismutase (SOD) activity, and malondialdehyde (MDA) levels were measured. Their lung aquaporin 1 (AQP1) and Occludin protein levels were analyzed.

**Results:**

LPS administration significantly decreased the ratios of PaO_2_/FiO_2_ and pressure-volumes and induced lung inflammation and injury by increased concentrations of TNF-*α*, IL-6, IL-10, and TP in BALF and MPO and MDA in the lung but decreased PC in BALF and SOD activity in the lungs. LPS also reduced AQP1 and Occludin protein levels in the lung of rats. In contrast, ACE significantly mitigated the LPS-induced lung injury, inflammation, and oxidative stress and preserved the AQP1 and Occludin contents in the lung of rats.

**Conclusions:**

ACE significantly improved respiratory function by mitigating inflammation and oxidative stress and preserving AQP1 and Occludin expression in the lung in a rat model of LPS-induced ARDS.

## 1. Introduction

Pulmonary infection with Gram-negative bacteria or inhalation of lipopolysaccharide (LPS) can cause lung injury, which can progress into acute respiratory distress syndrome (ARDS) with a high mortality. Currently, there is no effective treatment for ARDS in the clinic. Actually, the pathogenesis of ARDS is complex and remains unclear. Acupuncture is a traditional Chinese medicine, and because of its unique efficacy, acupuncture has been an alternative therapeutic strategy for many diseases in Western countries. Acupoint catgut embedding (ACE) is the extension and development of acupuncture by combining ancient traditional acupuncture with modern tissue therapy. The embedded absorbable suture in an acupoint acts as a durable stimulus, and it undergoes softening, liquefaction, and absorption in the acupoint. These, together with its easy operation and long-term effects, make ACE as an effective therapeutic strategy for weight loss, asthma, and rheumatoid arthritis in the clinic [1–4].

Previous clinical studies have demonstrated that ACE therapy can improve the pulmonary function in patients, who have chronic obstructive pulmonary disease (COPD) or bronchial asthma [5, 6]. A recent study has shown that the therapeutic efficacy of ACE is superior to manual acupuncture in improving functional mobility in patients with cerebral infarction [7]. Acupuncture or electroacupuncture at Zusanli is effective to control inflammation in rodents [8–11]. In addition, other studies have also revealed that electroacupuncture at Zusanli and Feishu can improve lung function and has anti-inflammatory effects in an endotoxin shock model [12–14]. Moreover, ACE exhibits analgesic effects on inflammatory pain induced by Complete Freund's Adjuvant in animals [15]. However, there is no information on whether and how ACE at bilateral Zusanli (ST36) and Feishu (BL13) can modulate the LPS-induced ARDS. Accordingly, we examined the therapeutic effects and potential action of ACE at ST36 and BL13 in LPS-induced ARDS in rats.

## 2. Materials and Methods

### 2.1. Animals and Groups

The experiments were approved by the Ethics Committee of Harbin Medical University. Male Sprague-Dawley (SD) rats (age between 8 and 9 weeks and weighs between 240 and 260 g) were provided by Harbin Medical University. All rats were housed in a specific pathogen-free room with a cycle of 12/12 h light-dark at a constant room temperature of 22°C to 24°C. All rats were assigned randomly into three groups each containing 20 rats: (1) the normal saline (NS) group, (2) lung injury (LPS) group, and (3) lung injury+ACE (LPS+ACE) group.

### 2.2. ACE Treatment

All rats were anesthetized intraperitoneally with 6% chloral hydrate (4 mL/kg body weight). After being shaved, their skin was disinfected with iodophor at the acupoint sites. The rats in the LPS+ACE group were implanted with a 2-3 mm absorbable suture (polyglycolide-co-lactide, Shanghai Tianqing Biomaterials, Shanghai, China) into the acupoints of bilateral Feishu (BL13, 3 mm lateral to the third thoracic vertebrae on the back, straight acupuncture, 8 mm) and Zusanli (ST36, 5 mm lateral to the anterior tubercle of the tibia, straight acupuncture, 7 mm) using a catgut embedding needle. The specific locations and acupuncture depths of the acupoints were determined using the Rat Acupoint Atlas. If the suture implantation was not successful or part of the suture remained outside of the skin, a new suture was implanted. The rats in the NS and LPS groups were given with the same doses of anesthetic and operated with a catgut embedding needle but did not receive a suture implantation. Rats were awake and freely moving within 60 minutes after the implanting procedure.

### 2.3. Model Establishment

Twenty-four hours after the ACE treatment, we established a rat model of LPS-induced ARDS, as a previous report [16]. Briefly, we anesthetized animals by intraperitoneal injection with 3% pentobarbital sodium (100 mg/kg), performed tracheostomy, and intubated them with a 16-gauge catheter connected to a ventilator (Harvard 7025, Ugo Basile, Varesse, Italy). We ventilated them with 1.0 FiO_2_, 8 mL/kg Vt, 1 : 2 inspiratory to expiratory ratio, and 2 cmH_2_O positive end-expiratory pressure (PEEP). We adjusted their breath rates to maintain an arterial 35-45 mmHg of partial pressure of carbon dioxide (PaCO_2_) and cannulated the femoral artery to monitor pressure (Datex, Helsinki, Finland) and to analyze blood gases (Bayer, Medfield, MA, USA). After the femoral vein was cannulated, the rats were injected intravenously with pancuronium bromide (1 mg/kg, Astra Zeneca, Bedfordshire, UK) and continually infused with pancuronium bromide (0.4 mg/kg^/^h). The rats were maintained by continuous intraperitoneal infusion with pentobarbital sodium (30 mg/kg^/^h) on a heat pad during ventilation. After stabilization for 10 min, their blood samples were collected from the femoral artery for measuring arterial blood gases hourly. The rats were instilled intratracheally with normal saline (NS group) or 0.5 mg/kg LPS (O55:B5, L2880, Sigma-Aldrich, USA) in 100 *μ*L saline (50 *μ*L each side of the lung in the LPS and LPS+ACE groups when the rats were at left and right decubitus positions), followed by a 3 mL air bolus in the trachea and ventilation for 2 min. The rats were continually ventilated for 4 h.

### 2.4. Specimen Collection

The animals were sacrificed. Subsequently, the right lung hilum was doubly liga-clipped, and the left lung was lavaged three times with 2 mL cold saline each. The bronchial alveolar lavage fluid (BALF) of individual rats was centrifuged, and their supernatants were stored at -80°C. The upper section weights of the right lungs were measured before and after drying in an oven at 80°C for 3 days to calculate the wet to dry (*W*/*D*) weight ratios. The middle section of the right lung was fixed in 4% paraformaldehyde, paraffin-embedded, and stained with hematoxylin and eosin, followed by examination under a light microscope. The degrees of lung injury in each sample were evaluated by histologists blindly and scored, according to infiltration or aggregation of inflammatory cells and thickness of the alveolar wall or alveolar collapse. Each assessment was graded as 0, appears normal; 1, mild damage; 2, moderate damage; and 3, severe damage. We calculated the total score for each animal [17]. In addition, we have frozen the remaining lung tissues at -80°C.

### 2.5. Static Compliance in the Left Lung

We measured pressure-volume (*P*‐*V*) curves of the left lung of each animal [18]. The airway pressure was raised stepwise from 0 to 30 cmH_2_O with 2 min of stress relaxation at each 5 cmH_2_O level and lowered in a similar fashion to 0 cmH_2_O. The lung volumes under different pressures were recorded, and left lung static pressure-volume (*P*‐*V*) curves were drawn.

### 2.6. Inflammatory Measures

We employed specific kits to measure TNF-*α*, IL-6, IL-10, and phosphatidylcholine (PC) concentrations in the BALF samples by an enzyme-linked immunosorbent assay (ELISA) (Meilian Biotechnology, Shanghai, China). The concentrations of total proteins (TP) in the BALF samples were determined using the BCA protein estimation kit (Beyotime, Haimen, China).

### 2.7. Detection of Oxidative Stress in the Lung

The lower lube of the right lung was homogenized in normal saline buffer to generate 10% lung tissue homogenates, and their protein concentrations were determined using the BCA protein estimation kit (Beyotime). The myeloperoxidase (MPO) activity, malondialdehyde (MDA) concentrations, and superoxide dismutase (SOD) activity in individual lung tissue homogenates were quantified using the appropriate detection kits (Jiancheng Biotechnology, Nanjing, China), according to the manufacturer's instructions.

### 2.8. Western Blotting Analysis of AQP1 and Occludin Protein Expression in Lung Tissues

Individual lung tissues (1 g each) were homogenized in 100 *μ*L radioimmunoprecipitation buffer (Beyotime) and centrifuged. After measuring protein concentrations, these samples were analyzed for AQP1 and Occludin protein levels by Western blot [19, 20] using rabbit polyclonal antibodies against AQP1 (1 : 5000, Abcam), Occludin (1 : 5000, Abcam), and *β*-actin (1 : 5000, Abcam). We quantified them using ImageJ software.

### 2.9. Statistical Analysis

Data are expressed as the mean ± standard deviation (SD). Difference among multiple groups was analyzed by one-way analysis of variance (ANOVA) or Kruskal-Wallis ANOVA and post hoc Student-Newman-Keuls test using SPSS 20.0. Statistical significance was defined when a *P* value < 0.05.

## 3. Results

### 3.1. ACE Mitigates Lung Damages in a Rat Model of LPS-Induced ARDS

**Figure 1:**
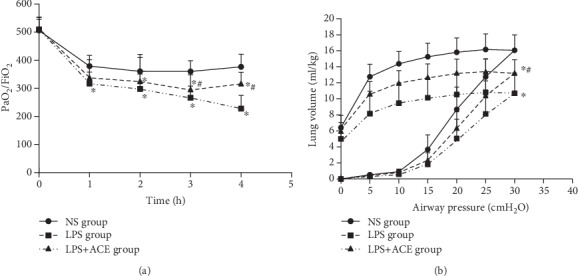
ACE improves the lung function. Following LPS instillation, the ratios of PaO_2_/FiO_2_ and pressure-volumes in individual rats were tested longitudinally at the specified time points. Data were the mean values ± SD of each group (*n* = 10 per group). (a) The ratios of PaO_2_/FiO_2_ in rats. (b) Pressure-volume (*P*‐*V*) curves. ^∗^*P* < 0.05 vs. the NS group; ^#^*P* < 0.05 vs. the LPS group.

**Figure 2:**
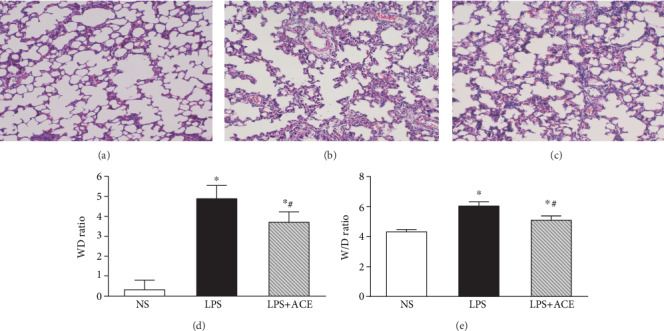
ACE mitigates the LPS-induced lung damage and edema in rats. Four hours after LPS instillation, the lung tissues from individual rats were subjected to hematoxylin and eosin staining and the severity of lung injury was scored. Furthermore, the wet/dry lung tissue weights were measured. Data are representative images (magnification ×100) or expressed as the mean ± SD of each group (*n* = 7) from two separate experiments: (a) the NS group; (b) the LPS group; (c) the LPS+ACE group; (d) quantitative analysis of lung injury scores; (e) the *W*/*D* lung weight ratios. ^∗^*P* < 0.05 vs. the NS group, ^#^*P* < 0.05 vs. the LPS group.

We detected the ratios of PaO_2_/FiO_2_ in all animals before and after intratracheal instillation with LPS longitudinally (Figure 1(a)). Compared with that before administration with LPS, LPS administration for 1 h significantly decreased the ratios of PaO_2_/FiO_2_ in rats (*P* < 0.05), while ACE treatment significantly increased the ratios of PaO_2_/FiO_2_ in rats at 3 and 4 h post LPS administration, relative to the LPS group (*P* < 0.05). Further measurement of the *P*‐*V* curves indicated that the volumes of *P*‐*V* curves in the LPS+ACE group were significantly smaller than those in the NS group, but larger than that of the LPS group (*P* < 0.05 for all, Figure 1(b)). At a pressure of 30 cmH_2_O, the values were 16.0 ± 1.9 mL/kg, 10.7 ± 2.5 mL/kg, and 13.2 ± 1.6 mL/kg in the NS, LPS, and LPS+ACE groups, respectively. In addition, histological examination revealed that a normal lung tissue structure displayed in the NS group of rats (Figure 2(a)) while there was severe lung injury, including interstitial and intra-alveolar edema and interalveolar septal thickening, alveolar collapse, and inflammatory cell infiltration, in the lungs of the LPS group of rats (Figure 2(b)). However, the degrees of lung injury in the LPS+ACE group of rats were obviously reduced, compared with those in the LPS group (Figure 2(c)). Quantitative analysis indicated that compared with those in the NS control, the lung injury scores in the LPS+ACE group significantly increased but remained significantly lower than those in the LPS group (*P* < 0.05 for all, Figure 2(d)). Moreover, a similar pattern was observed in the ratios of lung *W*/*D* weights among these groups of rats (Figure 2(e)).

### 3.2. ACE Mitigates the LPS-Induced Inflammation and Oxidative Stress in Rats

**Figure 3:**
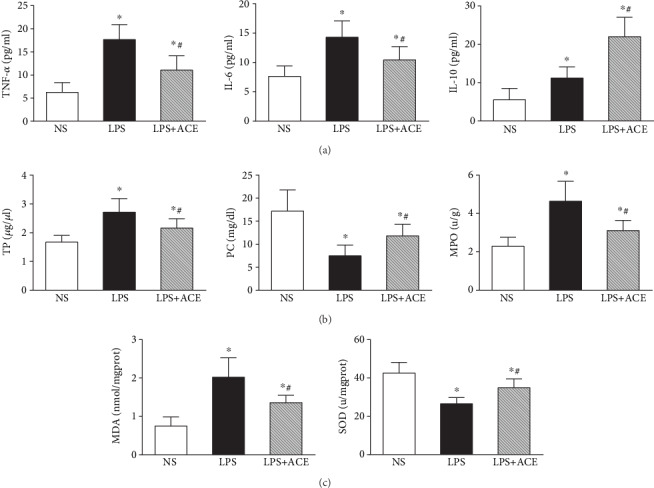
ACE mitigates the LPS-induced inflammation and oxidative stress in the lung of rats. Cytokine concentrations, PC and TP contents in the BALF, and MPO and MDA contents and SOD activity in the lung tissues of each animal were quantified. Data are expressed as the mean ± SD of individual groups (*n* = 10 per group) from three separate experiments: (a) cytokine concentrations; (b) TP, PC, and MPO levels; (c) MDA and SOD levels. ^∗^*P* < 0.05 vs. the NS group, ^#^*P* < 0.05 vs. the LPS group. TNF-*α*: tumor necrosis factor-*α*; IL: interleukin; TP: total proteins; PC: phosphatidylcholine; MPO: myeloperoxidase; MDA: malondialdehyde; SOD: superoxide dismutase.

To explore the potential mechanisms underlying the action of ACE, we measured the concentrations of inflammatory cytokines in BALF samples of all animals. Clearly, LPS administration significantly increased the concentrations of BALF TNF-*α*, IL-6, and IL-10 in animals (*P* < 0.05), and ACE treatment reduced the LPS-stimulated TNF-*α* and IL-6 production but enhanced IL-10 production in animals, relative to that in animals with LPS alone (all *P* < 0.05, Figure 3(a)). Similarly, LPS administration also increased the TP levels in the BALF and MPO in the lung tissues but decreased PC in the BALF of animals (*P* < 0.05 for all, Figure 3(b)). ACE treatment significantly mitigated the LPS-increased TP contents in the BALF and MPO in the lungs but elevated the PC in the BALF of rats, compared with that in the animals with LPS alone (all *P* < 0.05). In addition, LPS administration significantly enhanced the MDA levels but reduced the SOD activity in the lung tissues of animals while ACE treatment significantly mitigated the LPS-altered MDA levels and SOD activity in the lungs, relative to that in the animals with LPS alone (all *P* < 0.05, Figure 3(c)).

### 3.3. ACE Mitigates the LPS-Reduced Expression of AQP1 and Occludin Proteins in Animals

**Figure 4:**
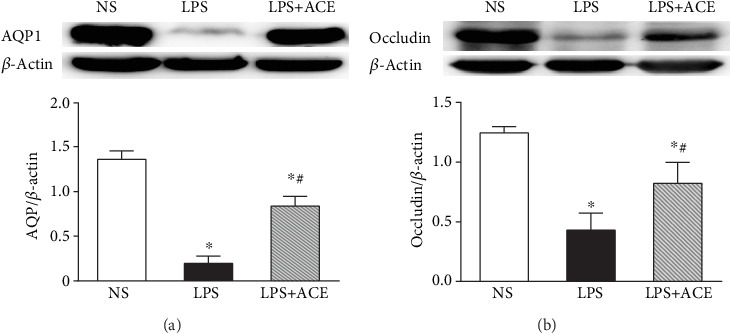
ACE mitigates the LPS-downregulated AQP1 and Occludin proteins in the lung tissues. AQP1 (a), Occludin (b), and *β*-actin proteins in the lung tissues of each animal were quantified by Western blot. Data are representative images or expressed as the mean ± SD of individual groups (*n* = 5 per group) from three separate experiments. AQP1: aquaporin 1. ^∗^*P* < 0.05 vs. the NS group; ^#^*P* < 0.05 vs. the LPS group.

We employed Western blot to quantify the expression of AQP1 and Occludin proteins in the lungs of all animals. We observed that LPS injection significantly decreased the expression of AQP1 and Occludin proteins and ACE treatment significantly mitigated the effect of LPS on decreased AQP1 and Occludin expression in the lung tissues of animals, compared with those with LPS alone (all *P* < 0.05, Figure 4).

## 4. Discussion

ARDS is characterized by a sudden onset, impaired gas exchange function, decreased lung compliance and pulmonary edema [21, 22], and an increase in pulmonary capillary permeability [23]. It is important to use an appropriate ARDS animal model for investigating the pathogenesis and potential therapies of ARDS [24]. Previous studies have shown that intratracheal instillation of LPS into animals can induce local inflammation and multiorgan failure syndrome without causing severely systemic inflammation in rodents, including rats [25–30]. Actually, intratracheal instillation of LPS at 0.1-8.0 mg/kg in rats can cause lung injury at 4-12 hours post induction [31]. In this study, we used intratracheal instillation with 0.5 mg/kg LPS to establish a rat ARDS model, and we found that LPS administration decreased lung function by gradually reducing PaO_2_/FiO_2_, increasing wet/dry lung weights and pathological changes and inflammation in the lung of rats. Evidently, we detected significantly higher concentrations of proinflammatory cytokines and TP in the BALF and MPO and MDA in the lung, but lower levels of PC and SOD, accompanied by decreased levels of AQP1 and Occludin, in the lung of rats. Such data indicate that LPS, through the Toll-like receptor 4 (TLR4), activates the NF-*κ*B signaling and causes oxidative stress and inflammation, leading to lung injury in rats. This rapid progression of lung injury was similar to that in patients with ARDS so that this model may be valuable for investigating the pathogenesis of ARDS and evaluating therapeutic efficacy of drug candidates for ARDS.

Previous studies have shown that ACE at BL13 and ST36 acupoints can significantly improve pulmonary function in the clinic [32, 33]. In this study, we found that ACE at BL13 and ST36 acupoints significantly mitigated the LPS-induced oxidative stress and lung injury and improved pulmonary function by inhibiting proinflammatory cytokine production and preserving AQP1 and Occludin expression in the lung of rats. Such data extended previous observations and indicated that ACE at BL13 and ST36 acupoints improved pulmonary function during the process of not only COPD or bronchial asthma but also ARDS.

Aberrant inflammatory responses are associated with the onset and development of ARDS, so that effective control of these inflammatory responses is one of the important strategies for the treatment of ARDS [34–36]. The BL13 and ST36 acupoints have been considered to regulate inflammation and immune responses [37, 38]. Intratracheal instillation of LPS can release a large number of proinflammatory factors by alveolar macrophages, lung capillary endothelial cells, and neutrophils. Among proinflammatory factors, TNF-*α* was a multifunctional cytokine and an important mediator in the early stages of an inflammatory response [39]. Other cytokines, such as IL-1*β*, IL-6, and IL-10, can be used for the diagnosis of sepsis and evaluating the inflammatory responses and the prognosis for ARDS patients [40]. As an inflammatory regulator, IL-10 can inhibit proinflammatory responses [41–43]. In the early stages of ARDS, pulmonary macrophages can produce various inflammatory factors, so that detection of inflammatory factors in the BALF was more accurate to reflect inflammation degrees. Neutrophils also play a key role in the early stages of ARDS. Actually, a pathological study has revealed a significant accumulation of neutrophils in the lungs [44], and many neutrophils are detected in BALF samples from ARDS patients [45, 46]. MPO is a sign of neutrophil aggregation and activation [47] and widely used as a marker of neutrophil counts and the severity of oxidative stress response [48]. Zhang et al. [14] reported that electronic acupuncture (EA) at Zusanli and Feishu improved lung function in rats with COPD by inhibiting inflammation. In our study, we found that ACE also decreased TNF-*α* and IL-6, but increased IL-10 concentrations in the BALF, and reduced lung tissue MPO activity in rats with ARDS. In contrast to the reduced production of TNF-*α* and IL-6, the increased IL-10 levels in the ACE group suggest that ACE may regulate immune response, and ACE can serve as an alternative strategy for vagal stimulation [49]. It is possible that ACE may activate the cholinergic anti-inflammatory pathway and enhance endogenous acetylcholine (ACh) production by immune cells to inhibit proinflammatory cytokine production. Actually, ACh can inhibit the production of LPS-induced IL-1*β*, IL-6, and IL-18, but not anti-inflammatory cytokine IL-10 [50].

Oxidative stress is a critical player in the occurrence and progression of ARDS. MDA and SOD are biomarkers and widely used to indicate the status of oxidative stress [51, 52]. Previous studies have showed that removal of excessive reactive oxygen species (ROS) can mitigate pulmonary damage caused by endotoxin [53]. Acupuncture at ST36 acupoint can resist septic shock in rats by activating the cholinergic anti-inflammatory pathways, significantly reducing inflammatory factor production, increasing anti-inflammatory factor production, inhibiting lipid peroxidation and oxygen free radical production, and mitigating organ damage [54, 55]. Furthermore, Zhang et al. [56] reported that acupuncture at ST36 and BL13 acupoints can mitigate the LPS-induced lung injury induced by endotoxic shock and significantly attenuate the LPS-increased MDA and TNF-*α*, as well as LPS-decreased SOD activity. In this study, we found that LPS administration significantly decreased SOD activity but increased MDA contents in lung tissues in animals. Such data indicated that ARDS induced by LPS caused oxidative stress in the lung. However, ACE at ST36 and BL13 acupoints significantly alleviated oxidative stress and improved lung function in rats.

The alveolar-capillary barrier facilitates efficient gas exchange and restricts the accumulation of fluid and large solutes in the alveolar space. When this barrier becomes dysfunctional, patients develop ARDS, which is clinically defined by a ratio of PaO_2_/FiO_2_ up to 300 mmHg [57]. In our study, using 100% oxygen inhalation, at 1 h post intratracheal instillation of LPS, PaO_2_ started descent, while at 4 h, PaO_2_ reduced to below 300 mmHg. These data were consistent with ARDS and showed oxygen dysfunction. However, ACE ameliorated the LPS-decreased PaO_2_ and improved pulmonary oxygenation at 4 h post intratracheal instillation of LPS in animals.

Pulmonary surfactant (PS) plays an important role in a broad range of treatments for ARDS. PS reduces surface tension and maintains lung volumes during the respiratory cycle. Lack of PS in ARDS causes atelectasis, leading to respiratory failure [58]. PS is mainly composed of a phospholipid-protein complex synthesized and secreted by alveolar type II epithelial cells, and phospholipids account for more than 90% and phosphatidylcholine (PC) for more than 50% of the phospholipids [59, 60]. Therefore, PC contents can mainly reflect the total amount of PS. In our study, the PC contents in BALF were significantly reduced and the TP concentrations in BALF markedly increased at 4 h post intratracheal instillation of LPS. The change in PC and TP concentrations in BALF indicated that the pathogenic process damaged the structure of alveolar type II epithelial cells and reduced PS contents in rats. However, ACE treatment mitigated the effect of LPS on the levels of PC and TP in rats. In addition, the *P*‐*V* curve represents the lung static compliance and is a hallmark of ameliorating lung physical function [18]. In this study, ACE also improved the *P*‐*V* curves and lung physical function.

The lung *W*/*D* weight ratio is a measure for the degrees of pulmonary edema, and it can assess lung vascular endothelial permeability [61]. In our study, we found that ACE treatment decreased the lung *W*/*D* weight ratios in rats, indicating that ACE alleviated the severity of pulmonary edema. Aquaporins (AQPs), a family of water channel proteins, are responsible for water movement between the airspace, interstitial, and capillary compartments [62]. AQP1, one member of the AQP family, is expressed in alveolar endothelium [63]. Tight junction (TJ) proteins can form paracellular channels to control the movement of water, solutes, and immune cells between both epithelial and endothelial cells [64]. The dysfunction of TJ proteins can lead to pulmonary barrier disruption and edema during the acute lung injury [65]. Occludin, the first member of the TJ family, is important for cell-cell connection [66]. Studies on Occludin have shown that multiple domains of Occludin modulate paracellular permeability [67, 68]. Therefore, AQP1 and Occludin functional impairments are associated with increased membrane permeability and edema [69, 70]. Previous studies have showed that AQP1 protein expression decreases significantly in LPS-induced acute lung injury [19, 71]. In our study, LPS administration significantly decreased the expression of AQP1 and Occludin proteins while ACE significantly mitigated the LPS-downregulated AQP1 and Occludin expression in the lungs of animals, extending previous studies [72, 73]. These data indicated that the preservation of AQP1 and Occludin protein expression in the lungs of LPS-treated rats by ACE alleviated pulmonary edema and improved vascular endothelial permeability in animals.

We recognized some inevitable limitations. This study had a short observation period, which may be insufficient to comprehensively evaluate the ARDS processes. Thus, our results mainly reflected the effect of ACE in the early process of LPS-induced ARDS. Furthermore, we only detected the effect of ACE at 1 day before the induction of lung injury; the long-term effect of ACE is needed to be observed. In addition, we did not investigate the molecular mechanisms by which ACE preserved the lung function. Therefore, further investigations are warranted.

## 5. Conclusions

The findings indicated that ACE BL13 and ST36 preserved the lung function and alleviated LPS-induced ARDS by improving pulmonary edema and pulmonary oxygenation, increasing lung compliance, and inhibiting inflammation and oxidative stress. These observations may provide a basis for design of treatments for ARDS. However, more studies are needed before clinical application can be considered.

## Data Availability

The data used to support the findings of this study are included within the article.
